# Call to action for global and national actors for open health data

**DOI:** 10.1371/journal.pdig.0000320

**Published:** 2023-08-08

**Authors:** Isabelle Rose I. Alberto, Nicole Rose I. Alberto, Alvin B. Marcelo

**Affiliations:** 1 College of Medicine, University of the Philippines, Manila, Metro Manila, Philippines; 2 Medical Informatics Unit, College of Medicine, University of the Philippines, Manila, Metro Manila, Philippines; 3 Department of Surgery, Philippine General Hospital, Manila, Metro Manila, Philippines; University Hospital Augsburg: Universitatsklinikum Augsburg, GERMANY

## Executive summary of open health data

Openness and sharing of discovery lie at the heart of the scientific method. Yet historically, the data and reports upon which they are based have not been easily accessible and often require onerous efforts to obtain. From this was borne the open data movement, which has advanced research and transformed public health policy-making in countries that have embraced this change. Open health data refers to unrestricted, free-of-charge access to health data [1]. By making health data available to all without any limitations to its reuse, it leverages national transparency, accountability, participation, innovation, and research through a multitude of data sources in large volumes—as well as paving the way for the creation of novel citizen-centric services [1,2]. The ability to access and combine diverse health data enriches analytical capacity and evaluation that can inform national policy development and improve healthcare delivery [3,4]. Open health data is therefore a public resource and should be delivered purposefully and proactively, in line with user needs and its potential contribution to value creation [2].

However, the open data movement comes with the need to balance its benefits with the security and privacy challenges that come with it, such as the risk of re-identification [4]. The issue of selling medical data to commercial organizations such as pharmaceutical companies, insurance companies, and other private companies has also stirred much controversy and raised concerns about ethicality, reproducibility, and generalizability [5]. Undeniably, challenges to the implementation of open health data remain, including the lack of awareness of its potential benefits, unsteady leadership and accountability, and weak legal and ethical oversight. Additionally, addressing the enormous gap in resources and knowledge on tools and techniques is acutely needed to make the technological shift to open and interoperable databases with unified protocols, frameworks, and terminology [3].

Despite the challenges in open health data, numerous countries and institutions have proven its feasibility with the proper legislation and implementation. By incorporating formal requirements in national open data strategies, laws, and regulations, the majority of Organisation for Economic Cooperation and Development (OECD) member countries have successfully adopted and scaled up “open by default” and “government as platform” approaches to data [2]. This has resulted in stronger governance frameworks and an increase in data availability, paving the way for the publication and use of open government data in various sectors geared towards social, government, and business innovation, with potentially important implications for public sector integrity, sustainable development, and gender equality [2]. With the passage of its e-government law, Germany has catapulted its data accessibility in recent years to provide data in machine-readable formats, with metadata descriptions, free of charge, and with unrestricted access for reuse [2]. Upon adoption of its Open Data Charter, Canada also released a number of online databases, such as The Public Health InfoBase and PulseNet Canada, which offer easy-to-use tools for accessing public health data on chronic diseases, mental health conditions, and foodborne molecular surveillance, to name a few [3]. Additionally, the widely utilized, freely-accessible critical care MIMIC databases have provided scientific opportunities for academic and industrial research, quality improvement initiatives, and higher education coursework [6–8]. Conversely, there is a critical need for the United Nations and other global organizations like the World Health Organization (WHO) to develop and espouse open health data as a global strategy as this movement consequently cascades to the regional and national levels.

## An approach to open health data

Making health data openly available is complex and requires the coordination and collaboration of many stakeholders. In such cases, a clear governance framework helps these actors to work together to achieve their common goal. One such framework is the Asia eHealth Information Network’s Mind the GAPS approach. AeHIN was created in 2011 by the WHO with the aim of strengthening digital development in Asian countries. GAPS is an acronym for Governance, Architecture, People and Program Management, and Standards–the four areas main areas that serve as enablers to further initiatives in open health data [9,10].

### 1. Governance: Decision-making and investment

Because digital health requires the coordination of numerous stakeholders and technology components, a clear governance framework is necessary to clarify responsibilities and accountabilities. This ensures there is a well-known leadership entity that oversees all constituencies and that sets the strategic direction for digital health in the country. This leadership entity establishes the shared goals and objectives of all the stakeholders and ensures that their activities contribute to the achievement of their collective goals. To achieve open health data, the leadership entity must include this explicitly in their national health data strategy.

While leadership structures and styles differ across cultures, adopting a governance framework ensures continuity over time and transparency among stakeholders. Fortunately, there are several information technology governance frameworks that can be adopted. One of the simplest is the Corporate Governance of Enterprise Information Technology (or ISO 38500) which lists six major areas that need to be covered. A more comprehensive framework is ISO TR 14639, which specifically describes the components of a whole national eHealth architecture. Straddling between these is COBIT 2019 (formerly Control Objectives for Information and Technology)—a “business framework for the governance and management of enterprise information technology.” COBIT 2019 specifies major processes for any sector adopting information technology, including health. Regardless of the choice of framework, all require the leadership entity to set a strategy upon which subsequent activities are based. It is therefore crucial, in the pursuit of open health data, for leaders to explicitly specify openness as part of the national digital health strategy.

Who should heed the call for an open data strategy? We opine that it needs to emanate at the global level—the United Nations (UN). With sectoral structures like the World Health Organization (WHO) for health and the International Telecommunications Union (ITU) for technology, there is a potential for the fragmentation of the open data strategy. And this fragmentation can cascade to the regions and countries. Thus, we call upon the UN to demonstrate multi-sector coordination and adoption of governance frameworks to model transformation at their level, espousing open health data as a global strategy. This is, in fact, already stated in the Global Strategy for Digital Health 2020–2025 [11]. If the open health data strategy is modelled at the global level, there is a higher chance for countries to adopt the same.

To ensure the proper cascade of the global open health data strategy, regional networks should be engaged in the formulation and design of policies at the international level. This maximize open health data benefits and minimize risks in the face of new data sources and technology [2]. The alignment of the UN, NGOs, WHO, and ITU in crafting open data policies at the global level is the key to regional and national alignments.

### 2. Architecture: Sharing a blueprint for all

This open health data strategy must be embedded in a shared architecture (or blueprint). According to The Open Group Architecture Framework (TOGAF), enterprise architecture can be defined in two contexts: (1) “a formal description of the system at component level to guide its implementation” and (2) “the structure of components, their inter-relationships, and the principles governing their design and evolution over time” [12]. Opening health data is a complex process, and the lack of an architectural blueprint impedes this undertaking. A blueprint clearly establishes the alignment of business processes, data, application, and technologies. It also depicts the security of this endeavor, reducing the hazards and risks associated with it. It defines accountability for each stakeholder, functions as a common reference point, and guides recognition of what needs to be established, strengthened, or modified. Moreover, since the effectiveness of the blueprint is limited by the compliance of involved stakeholders, proper identification and consideration of their risks, benefits, and resources should be done during development and expansion [9].

OpenHIE is one example of an architecture framework that can serve as a foundation for opening health data. It is based on individual publicly accessible software components inter-operating to consolidate and integrate all health information exchanges originating from various external systems and points of service into one unified record [13]. A solid and transparent healthcare enterprise architecture can optimize organizational digital health capabilities and effectively facilitate the shift to open health data and information technology [14].

### 3. People & program management: Certified architects and engineers who will be building the house

To achieve open health data, there is a need to invest in people and program management. Through this, the leadership entity can ensure the data science capability and capacity of stakeholders in building and maintaining their assigned components in the blueprint. This is especially important for key sectors that connect multiple components, such as registries and health information exchanges. A pool of skilled, engaged, and certified project managers and staff that are knowledgeable about enterprise architecture (e.g., TOGAF, OpenHIE) and project management (e.g., PRINCE2 [15], PMP [16]) must be established to effectively deliver the roster of open health data initiatives. Institutional readiness and adequate system assessment must be ensured as these serve as the groundwork for the implementation of policies and strategies for digital health [17].

Once the governance structure, architecture, and program management mechanisms are in place, private sectors are now empowered to contribute to the development of new technologies and the creation of interoperable health information systems. Global players and countries should discuss and cooperate on common needs through convergence workshops, as established by the AeHIN. At a national level, through convergence meetings among the government, development partners, and the private sector, the leadership of the ministry of health is established, and partnerships that strive to implement the open health data policy are fostered [10].

### 4. Standards and interoperability: Building blocks for open health data

Lastly, an essential requirement for the success of any open health data initiative is health information system interoperability [18]. Interoperability in the context of healthcare pertains to prompt and secure access, exchange, analysis, and integration of health information within and across institutional boundaries, and this is achieved partly using coherent system standards. These standards provide both a syntactic and semantic definition of health information and serve as the architectural foundation to be built up by stakeholders [18]. Hence, for effective open health data, standards regarding resources, indicators, data sources, data management, information products, dissemination, and use should be properly identified and promoted among stakeholders.

A network of competent interoperability professionals should be created to support the interconnection needs of government, academia, and private sector. The AeHIN Regional Enterprise Architecture Council for Health or REACH is a community of practice around interoperable health information systems. Countries and international agencies seeking interoperability should agree to publish and coordinate their blueprints, or else they will be producing open health data in silos.

## A Call to action

To harness the benefits of open health data, global and national actors must explicitly include open health data into their national strategy. This strategy must reflect the careful balance of freedom with regulation and of innovation with control. To create this environment, clear governance structures and frameworks must be established where responsibilities and accountabilities are understood and owned by stakeholders. By embedding open health data in the national digital health blueprint, the leaders will be creating trustworthy processes where the provenance of the data can be assured, allowing for research reproducibility and enabling peer review. To fully maximize open health data for global use, international agencies led by the United Nations are called to show leadership by aligning agendas and breaking long-standing silos between sectors. If this alignment cascades down to the regional and national levels and common standards are made available, it is possible to create a global open data repository that can be processed not just for health but can also be applied to finance, education, social welfare, agriculture, and other societal sectors.
